# The Influence of High-Dose Parenteral Vitamin C on the Incidence and Severity of Postoperative Pulmonary Complications in Cardiac Surgery with Extracorporeal Circulation: A Randomized Controlled Trial

**DOI:** 10.3390/nu16060761

**Published:** 2024-03-07

**Authors:** Milica Karadžić Kočica, Arsen Ristić, Ivan Soldatović, Dejan Lazović, Jelena Čumić, Miloš Grujić, Radmila Karan, Duško Terzić, Ivan Palibrk, Mladen Kočica, Dejan Marković

**Affiliations:** 1 Department of Anesthesiology, Reanimatology and Intensive Care, Clinic for Cardiac Surgery, University Clinical Centre of Serbia, 11000 Belgrade, Serbia; milica.karadzic@gmail.com (M.K.K.); jelena.cumic@gmail.com (J.Č.); karan.radmila@gmail.com (R.K.); drdejanmarkovic@gmail.com (D.M.); 2 Medical Faculty, University of Belgrade, 11000 Belgrade, Serbia; arsen.ristic@med.bg.ac.rs (A.R.); ivan.soldatovic@med.bg.ac.rs (I.S.); lazovic.dejan88@gmail.com (D.L.); drgrujicmilos@gmail.com (M.G.); terzic.dusko@gmail.com (D.T.); ivanpalibrk@yahoo.com (I.P.); 3 Clinic for Cardiology, University Clinical Centre of Serbia, 11000 Belgrade, Serbia; 4 Department of Medical Statistics and Informatics, Medical Faculty, University of Belgrade, 11000 Belgrade, Serbia; 5 Clinic for Cardiac Surgery, University Clinical Centre of Serbia, 11000 Belgrade, Serbia; 6 Department of Anesthesiology, Reanimatology and Intensive Care, Clinic for Abdominal Surgery, University Clinical Centre of Serbia, 11000 Belgrade, Serbia

**Keywords:** vitamin C, ascorbic acid, high-dose, parenteral administration, cardiac surgery, extracorporeal circulation, postoperative pulmonary complications

## Abstract

Cardiac surgery (CS) with extracorporeal circulation (ECC), induces intense oxidative stress (OS) and systemic inflammatory response (SIR), which may seriously affect postoperative lung function. We aimed to test if high parenteral (200 mg/kg/24 h) daily doses of Vitamin C (VitC), given within 48 h after the beginning of the operation, may reduce the incidence and severity of postoperative pulmonary complications (PPCs) in CS patients. This single-center, prospective, randomized, single-blinded, interventional trial included 150 patients, assigned to control Group A (*n* = 75) and interventional Group B (*n* = 75). Group B intraoperatively received one-fourth (i.e., 50 mg/kg) of the planned daily Vit C dose, divided into three equal parts and diluted in 10 mL of normal saline, while Group A received an equal volume of normal saline at the same time frames (i.e., the induction of anesthesia, aortic cross-clamp release, and sternal closure). After 6 h from the first intraoperative dose, the following regimen was applied: Group B: 50 mg/kg, 30 min i.v. infusion of VitC in 50 mL of normal saline, every 6 h, for the next 48 h, and Group A: 30 min i.v. infusion of an equal volume of normal saline every 6 h, for the next 48 h. Modified Kroenke’s score was used to determine the incidence and severity of PPCs. The overall incidence of PPCs was 36.7% and was significantly lower in Group B (13.3% vs. 60.0%, *p* < 0.001). The PPCs severity score was also significantly lower in Group B (1 vs. 3, *p* < 0.001). In addition, patients from Group B had significantly less damaged lungs, better postoperative renal function, shorter ICU stays, fewer ICU re-admissions, and lower hospital mortality. No VitC-related adverse effects were recorded. High parenteral daily VitC doses given within 48 h after the beginning of CS are safe and effective in reducing the incidence and severity of PPCs. A multicenter RCT is needed to confirm these results.

## 1. Introduction

Cardiac surgical (CS) operations with extracorporeal circulation (ECC), inevitably induce a complex, multi-etiological, oxidative stress (OS), and systemic inflammatory response (SIR), the intensity of which varies from mild, subclinical forms, to clinically manifest syndrome (SIRS), with or without multiorgan dysfunction (MODS) [1,2,3,4,5]. The incidence of these events in CS is higher compared to other elective surgeries [6,7].

A combination of different procedural stressors (anesthesia, perfusion, and surgery) is responsible for the initiation of SIR and OS, being the most intense within the first 24–48 h after CS operations. Different therapeutic strategies, aimed primarily at the control of certain inducing factors of inflammation (i.e., contact activation, hemodilution, hypothermia, endotoxemia, ischemia-reperfusion injury, and tissue damage), failed to reduce the systemic effect of numerous pro-inflammatory stimuli [2,4]. Anti-mediator therapy with monoclonal antibodies against selected pro-inflammatory cytokines also did not give satisfactory results. It has been experimentally and clinically established that Nuclear Factor kappa-B (NFκB), as a central transcription factor, determines the intensity and extent of systemic inflammation. Strategies aimed at the selective inhibition of NFκB did not prove useful, because they also prevent beneficial, reparative inflammation, thus causing greater tissue damage in places where the conditions for the development of inflammation normally exist. Strategies that reduce, but do not inhibit NFκB activity, (i.e., ischemic or thermal preconditioning, treatment with lipopolysaccharides, etc.) represent, for now, the optimal theoretical framework for controlling the postoperative inflammatory response, but so far it has been clinically confirmed only at the level of individual organs and tissues (e.g., myocardium) [3].

Identifying ECC as a key factor in the genesis of excessive SIR and OS in cardiac surgery, since the early eighties, the method of surgical myocardial revascularization without the use of ECC (Off-Pump CABG, OPCABG) has been perfected. Potential benefits of OPCABG over on-pump CABG (the avoidance of contact activation, hemodilution, hypothermia, less pronounced ischemia-reperfusion injury, blood transfusion, and preserved pulsatile flow) should be carefully measured against its shortcomings (incomplete revascularization, mechanical stress and shear forces on the heart, technical skills, specific anesthesia, and the release of inflammatory mediators from other sources) [8]. The evidence for the superiority of Off-Pump CABG over On-Pump CABG, in terms of inflammation and outcome, is still controversial and inconsistent. Only four out of ten RCTs were able to connect inflammatory suppression and better clinical outcomes [9,10]. The surgical trauma, which is significant and common in both revascularization strategies, and recent findings of early and excessive endothelial glycocalyx layer disruption and shedding during the OPCABG, is believed to be more important than ECC in terms of SIR and OS [8,11].

Within this continuum, the lungs are both the source and target organ for oxidative and inflammatory mediators [3,4,5,12]. Accordingly, postoperative pulmonary complications (PPCs) are the most frequent complications of CS interventions with ECC and are responsible for significant morbidity, disability, mortality, and health care costs [13,14,15]. The length of hospital stay for these patients is prolonged by 13–17 days, and healthcare costs are increased by 41–47%. The patients suffering from PPCs are at significant risk for both early (14–30%) and late (one-year and five-year) mortality (45.9% and 71.4%, respectively) [13]. The incidence of PPCs ranges from 2% to 90%, according to different criteria for their definition [14,16,17,18]. The European joint taskforce for Perioperative Clinical Outcome (EPCO) published guidelines in 2015, proposing seven components be considered as a composite outcome measure for PPCs: respiratory infection, respiratory failure, pleural effusion, atelectasis, pneumothorax, bronchospasm, and aspiration pneumonitis [19]. Three years later, the Standardized Endpoints for Perioperative Medicine (StEP) collaboration task force proposed four components, sharing the same biological mechanism (i.e., pulmonary collapse and airway contamination) to be a composite outcome measure for PPCs: atelectasis, pneumonia (CDC definition), ARDS (Berlin definition), and pulmonary aspiration [16]. Despite ongoing efforts to establish a widely recognized, standardized definition of PPCs as a composite outcome measure, this task has not been accomplished so far [13]. Modified Kroenke’s (Supplementary Materials S1, Table S1) severity score (grades 0 to 5) with operational definitions of PPCs included, combines diagnostic criteria from both above-mentioned guidelines (i.e., atelectasis, bronchospasm, pleural effusion, pneumonia, pneumothorax, and respiratory failure), in a way suitable for both clinical practice and trial analyses [20,21,22,23].

Among different strategies aiming to attenuate SIR and OS, the possible role(s) of vitamin C (VitC, ascorbic acid, ascorbate) seems to be underestimated. Thanks to its proven antioxidant and pleiotropic biochemical functions, and its ability to reduce the intracellular activation of NFκB, which determines the intensity and extent of systemic inflammation, vitamin C (VitC, ascorbic acid, ascorbate) is increasingly used in various conditions to reduce an excessive OS and SIR [3,24,25,26,27,28,29,30,31,32,33]. Yet, the optimal dose, timing, and route of VitC administration are still unclear [34].

It was shown that patients undergoing non-CS procedures (i.e., those not directly involving the heart and its great vessels) need much more VitC (500 mg–4000 mg/24 h) than the recommended daily doses for healthy individuals (90 mg/24 h) [29,35,36]. Cardiac surgical patients are even bigger and faster VitC “consumers” [34], but, despite this, the majority of them (56%) enter CS with VitC deficiency [29,37].

Since 1970 onward, the scientific community has been divided on the safety and efficacy of high-dose parenteral VitC supplementation [38,39]. Several studies conducted during the COVID-19 pandemic, the NIH expert panel document on cancer treatment, and studies on critically ill patients, have reported no adverse events with high daily parenteral doses of up to 50 g [40,41,42,43,44,45]. The most recent pharmacokinetic studies proved the safety of even higher parenteral doses of up to 100,000 mg/24 h [46,47].

In addition to well-documented safety, high parenteral doses of VitC were proven efficient in both surgical and non-surgical cardiac patients. Beneficial effects of this vitamin in non-surgical cardiac patients include the differentiation of stem cells into cardiac myocytes [48], cardioprotection (during ischemia-reperfusion) [49], an increase in vasopressor-sensitivity, and the improvement of endothelial function and coupling (the prevention of edema) [50,51]. In six RCTs exploring the effect of Vit C in non-surgical patients with low LVEF, an average increase of 12.0% was achieved, with daily doses from 1000–10,000 mg [52]. Cardiac surgical patients may have even more benefits from VitC supplementation [36,45]. The prevention of ischemic and oxidative myocardial damage [45], improvement of ventricular function, reduction of vasopressor and fluid demand (preventing vasoplegia), and reduced occurrence of postoperative arrhythmias have been well documented so far [36]. A meta-analysis of 19 RCTs with 2008 CS patients, found that high doses of vitamin C (1000–6000 mg/24 h) reduced the incidence of postoperative atrial fibrillation, acute kidney injury, mechanical ventilation duration, intensive care unit (ICU) length-of-stay, and hospital length-of-stay, but had no effect on mortality [34]. Its effectiveness in CS was tested in a relatively small number of studies, focusing mainly on postoperative atrial fibrillation [34,35,36,53,54]. The only study that examined the effect of VitC on PPCs in low-risk CS patients, was limited to intraoperative parenteral administration of intermediate therapeutic doses (3 × 1000 mg) and showed significantly lower incidence and less severe PPCs in the intervention group [55].

We present the results of our trial, designed primarily to investigate the influence of high parenteral (200 mg/kg/24 h) daily doses of VitC, given within 48 h after the beginning of the operation, on the incidence and severity of PPCs in patients undergoing CS with ECC. The rationale for this therapeutic regimen relied on the fact that OS and SIR are most intense within the first 48 h after CS, and so is the peak consumption of vitamin C [5,34].

## 2. Materials and Methods

### 2.1. Design

The prospective, randomized, single-blinded, interventional trial was conducted at the UC Clinical Centre of Serbia, Clinic for Cardiac Surgery, Belgrade, Serbia, from July 2022 to November 2022. The trial was conducted following the guidelines of the Declaration of Helsinki and CONSORT (Consolidated Standards of Reporting Trials) standards for randomized controlled trials (RCT) (Supplementary Materials S2 and S3). Ethical approval was given by the Ethics Committee of Medical Faculty UC Belgrade, Serbia (protocol code 1322/VII-24). The trial was registered at the International Standard Randomised Controlled Trial Number (ISRCTN) registry (identifier: ISRCTN29876186). Informed consent was obtained from all participants, who also consented not to be informed about randomization and grouping.

### 2.2. Participants

Inclusion criteria were as follows: all patients aged ≥18 years undergoing an elective CS procedure with ECC, regardless of the type of planned operation. Exclusion criteria were as follows: previous CS operation, emergency patients, clinically and/or radiographically active lung disease, systolic pressure in the pulmonary artery >60 mmHg, allergy to ascorbic acid, gout, hemodialysis, significant oxaluria, uric nephrolithiasis, glucose-6-phosphate dehydrogenase enzyme deficiency, hemochromatosis, sickle cell anemia, sideropenic anemia, and thalassemia.

The criteria for subsequent withdrawal from the trial were as follows: operations with an ECC time ≥6 h, death during hospitalization caused by non-pulmonary reasons, and the withdrawal of previously given consent to participate in the trial.

### 2.3. Interventions

All patients were operated on and subsequently treated according to standard institutional anesthesiological, surgical, and intensive care protocols.

The protocol for VitC administration (Supplementary Materials, Figure S4) is original and was designed for this trial by compiling different regimens and positive experiences from the available literature [25,29,34,35,44,45,55,56].

Patients from the intervention group (B) received 200 mg/kg of Vit C daily (i.e., 50 mg/kg/6 h) during the first 48 h from the beginning of CS (i.e., a total of 400 mg/kg/48 h). The first dose (50 mg/kg), given intraoperatively, was further divided into three equal parts, diluted in 10 mL of normal saline, and administered via a central venous catheter in three different time frames: 10 min after the induction of anesthesia, 10 min before the removal of the aortic cross-clamp (reperfusion), and at the beginning of sternal closure. The control group (A) intraoperatively received an equal volume of normal saline at the indicated time frames.

Postoperatively (starting 6 h after the first intraoperative dose), VitC was administered according to the following regimen, for the remainder of the 42 h:

- Intervention group (B): 50 mg/kg/6 h as a 30 min i.v. infusion of VitC in 50 mL of normal saline every 6 h, under UV protection.
- Control group (A): an equal volume of normal saline every 6 h as a 30 min i.v. infusion, under UV protection.

The day after parenteral administration was ceased, patients from Group B continued to receive an enteral supplementation of VitC (2 g/24 h) until discharge, and were advised to continue it for a week after (Supplementary Materials S1, Figure S4).

For each patient included in the trial, standard perioperative characteristics (i.e., demographic, anthropometric, clinical, and laboratory) were collected from the medical records and entered in a separate database. The American Society of Anesthesiologists (ASA) score was calculated to assess the preoperative physical status [57]. The degree of organ dysfunction after surgery was quantified by the Sequential Organ Failure Assessment (SOFA) score which was estimated 48 h after CS [58].

The incidence and severity of PPCs were scored and agreed upon by two independent, blinded assistants, using the original operational definitions of Kroenke [23] and subsequent modifications [20,21,22,59]. Score values were determined daily, and as an individual PPK severity score, the worst registered value (i.e., the highest grade) within 7 days after surgery was used for the analyses. The severity of PPCs was graded on an ordinal scale of 0 (no PPC) to 5 (death before discharge). The values from 1 to 4 depict the increasing severity of the PPCs (Supplementary Materials S1, Table S1). To determine the incidence of PPCs, only scores ≥3 were considered.

Parameters indicating postoperative oxygenation and ventilation, selected inflammatory markers, and parameters of renal function were analyzed 48 h after surgery when the OS and SIR are expected to be the most intense [5,34].

### 2.4. Outcome

The primary end-point was to compare the incidence and severity of PPCs in the control (A) and intervention (B) groups.

Secondary end-point measures were grouped to compare the following: pulmonary oxygenation and ventilation (Horowitz index: PaO_2_/FiO_2_; alveolar–arterial gradient: A-aDO_2_; and time spent on mechanical ventilation), inflammatory markers (procalcitonin, C-reactive protein, leucocytes, neutrophils, lymphocytes, sedimentation rate, fibrinogen, albumin, D-dimer, and ferritin), renal function (GFR < 60 mL/min, creatinine, and urea), non-pulmonary postoperative complications, postoperative organ dysfunction (SOFA score and ASA/SOFA ratio), intensive care unit (ICU) parameters (re-admission and length of stay) and hospital parameters (length of stay and mortality) between the groups.

### 2.5. Sample Size

The required number of subjects to be included in the trial was calculated based on the primary objective—the assumption that high daily doses of parenterally administered VitC will reduce the severity of PPCs after CS with ECC. To provide a statistical power of 0.90, 5% level of significance, and to detect a clinically significant difference in the mean value of the PPC score ≥0.3 (2.1, 95% CI, 2.0–2.3 vs. 1.8, 95% CI, 1.7–2.0), as defined by previous studies [20,55], the required sample size was 70 subjects in each group (i.e., a total of 140). To allow for 15% drop-out, we decided to include a minimum of 80 participants in each group (i.e., a total of 160).

### 2.6. Randomization and Masking

Randomization was done by allocating each consecutive respondent in a 1:1 manner to either control (A) or intervention (B) groups, the order of which was determined by manual random selection. Except for the principal investigator (M.K.K.) and two assistants (J.Č., R.K.), nobody else involved in the patient treatment, clinical and laboratory data collection, and analysis (statistician) had any information about the randomization and grouping. Two informed assistants prepared an unlabeled infusion solution (placebo or VitC), covered it with a UV protective coating, and assigned it to the patient according to randomization and grouping. The principal investigator and two informed assistants were not engaged in the patient treatment, clinical and laboratory data collection, and analysis. Two independent, blinded specialists estimated and agreed on the ASA, SOFA, and PPC severity score grades. It was not feasible to meet the full criteria for a double-blinded trial, due to organizational and technical reasons, so the selective masking we applied was the best we could do to reduce the risk of bias and confounding.

### 2.7. Statistical Methods

Results are presented as count (%), means ± standard deviation, or median (inter-quartile range) depending on data type and distribution.

Groups were compared using parametric (*t*-test) and nonparametric (Chi-square, Mann-Whitney U test, Fisher’s Exact test) tests. Numerical data with normal distribution were compared using a *t*-test, while numerical data with non-normal distribution were compared using the Mann-Whitney U test. Data distribution was analyzed using statistical tests for normality, graphical methods (histogram, boxplot, Q-Q plot), and descriptive statistics. Ordinal data were analyzed using the Mann-Whitney U test. Nominal data were analyzed using the Pearson chi-square test to see if assumptions were met. If not, Fisher’s exact test was used instead. Logistic regression analysis assessed the significant correlation between binary outcome variables and treatment, with and without adjustment.

All *p*-values less than 0.05 were considered significant.

To conduct all statistical analyses, we used SPSS 29.0 (IBM Corp. Released 2023. IBM SPSS Statistics for Windows, Version 20.0. Armonk, NY, USA: IBM Corp.) and R 3.4.2. (R Core Team (2017). R: A language and environment for statistical computing. R Foundation for Statistical Computing, Vienna, Austria. URL: https://www.R-project.org/, accessed on 17 October 2023).

## 3. Results

**Figure 1:**
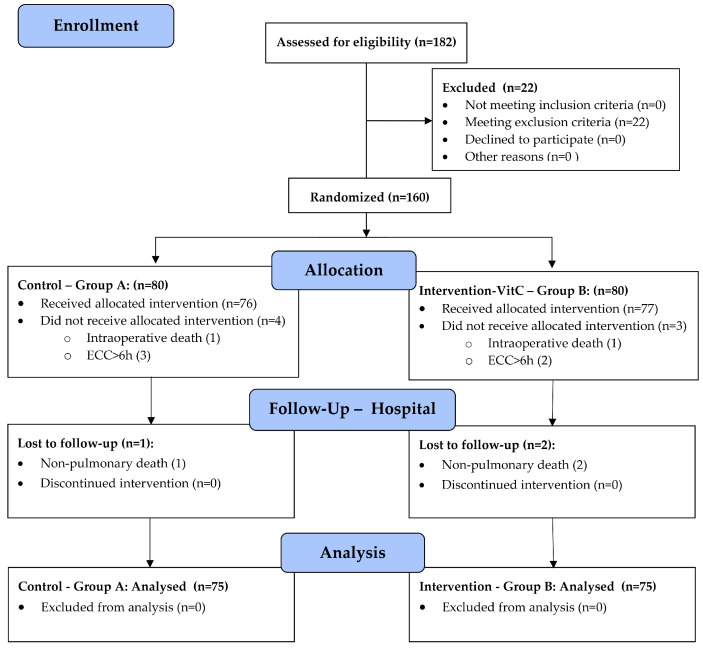
CONSORT (Consolidated Standards of Reporting Trials) flow diagram for the trial. (See Supplementary Materials S2—CONSORT Flow-Diagram, and Supplementary Materials S3—CONSORT check-list for the RCT).

After assessing for eligibility, 22 of 182 enrolled patients met the exclusion criteria and were not included in randomization. A total of 160 patients were randomized and allocated to control group—A (*n* = 80) and intervention (VitC) group—B (*n* = 80). A total of 10 patients were subsequently excluded from the analysis. In Group A, one was excluded for intraoperative death, three for ECC duration of ≥6 h, and one for in-hospital non-pulmonary death. In Group B, one was excluded for intraoperative death, two for ECC duration of ≥6 h, and two for in-hospital non-pulmonary death. As a result, a total of 150 patients (75 in each group) were available for the trial analyses (Figure 1, Supplementary Materials S2). They were followed up until hospital discharge.

The baseline perioperative characteristics of patients included in the trial are shown in Table 1. There were no significant differences between the groups for most of the perioperative parameters, except for body mass index (BMI), diastolic arterial pressure (TAd), chronic renal failure (CRF), and duration of surgery, which showed significantly higher values in Group A.

Although the other characteristics are fairly evenly distributed between the groups, it is evident that all patients had advanced systemic disease (ASA scores 3 and 4). Both groups were elderly people with pronounced CVD risks (HTA 97.3% in Group B and 100% in Group A) and serious comorbidities. Interestingly, the proportion of patients with a history of COVID-19 infection was relatively small, keeping in mind that this trial was conducted in a critical period (March 2020 to November 2022), when most of the infected and deceased were recorded [60]. Yet, preoperative pulmonary status in both groups was quite good (PPC score grades 0 and 1).

The primary end-point outcome measures of patients included in the trial are shown in Table 2. The overall incidence of PPCs was 36.7% and was significantly lower in Group B (13.3% vs. 60.0%, *p* < 0.001). The severity of PPCs was also significantly lower in Group B [1(1) vs. 3(2), *p* < 0.001]. Logistic regression analysis reveals significantly lower chances of PPC ≥ 3 in Group B [OR = 0.468 (95% CI 0.357–0.613); *p* < 0.001]. When adjusted for confounding factors, such as BMI, TAd, CRF, and duration of surgery, there is still a significant difference between groups regarding PPCs incidence [OR = 0.521 (95% CI 0.379–0.714); *p* < 0.001].

The secondary end-point outcome measures are shown in Table 3. The following parameters were significantly better in Group B: Horowitz index (312.6 ± 107.4 vs. 268.9 ± 112.6, *p* = 0.008), C-reactive protein [95 (56.2) vs. 167.4 (82.9), *p* < 0.001], sedimentation rate [20 (6) vs. 22 (18), *p* = 0.023], acute renal failure (1.3%vs. 10.7%, *p* = 0.034), wound infection (6.7% vs. 20%, *p* = 0.016), GFR < 60 mL/min (13.3% vs. 32%, *p* = 0.006), urea (6.3 ± 1.9 vs. 7.1 ± 3, *p* = 0.041), ICU re-admission (5.3% vs. 20%, *p* = 0.007), ICU stay [32 (24) vs. 48 (24), *p* < 0.001], and hospital mortality (1.3% vs. 10.7%, *p* = 0.034). Serum procalcitonin levels were significantly lower in Group A [0.3 (0.6) vs. 0.5 (0.8), *p* = 0.032].

## 4. Discussion

The results of our trial support the parenteral administration of high daily doses of VitC (200 mg/kg/24 h for 48 h) to reduce the incidence and severity of PPCs after CS with ECC (see Table 2). In addition, patients from the VitC group had significantly less damaged lungs (i.e., better Horowitz index), better postoperative renal function, shorter ICU stay, fewer ICU re-admissions, and lower hospital mortality (see Table 3).

In trying to find the answers to the primary end-points of this trial, we uncovered a question: “Do we know what is meant by PPCs in CS?” A simple query of “postoperative pulmonary complications” in PubMed retrieves 2155 articles published since 1927 (1322 published during the last 10 years). By adding “cardiac surgery” into the query, a total of 171 articles since 1976 are retrieved. Among them, at least 20 different definitions of PPCs can be found, varying in terms of the criteria, the timing, and the severity. None of them were the same or sufficiently similar to allow robust comparisons [14,17,61,62,63,64,65,66]. By comparing the guidelines issued by two respectable task forces [16,19], one can find seven listed complications used as a composite outcome measure in one definition of PPCs [19] and four in another definition [16] of PPCs. To overcome this, we chose the operational definitions of PPCs by Kroenke et al. [20,21,22,23], (Supplementary Materials S1, Table S1) since they combine diagnostic criteria from both reporting guidelines in a way that was the most suitable for our clinical practice and analysis. However, is it just a lack of a standardized definition of PPCs that is the problem? Some believe that the disparities in the clinical appraisal of PPCs are even more important [67,68]. Accordingly, the incidences of PPCs reported in the literature range widely from 2% to 90% [14,16,17,18,63]. The overall incidence of PPCs in our trial (36.7%), fits in the lower part of this range.

Another seemingly simple question was the following: “If it’s not severe enough, is it a complication at all?” Indeed, a certain degree of PPCs affects almost all CS patients, varying from mild, subclinical forms (e.g., compensated abnormalities of respiratory mechanics) to severe respiratory failure with prolonged ventilator dependency [69]. Reviewing the literature, as for the definition of PCCs, we could find very few reports defining standardized and objective criteria for the quantification of the severity of PPCs in CS [16,20,21,22,23]. Modified [20,21,22,23] Kroenke’s PPCs severity score offered an acceptable and applicable grading system for the severity of PPCs (Supplementary Materials S1, Table S1). It was recently compared with the Melbourne Group Scale, and both were deemed to be useful tools for grading PPCs in CS patients [59]. To overcome Kroenke’s score inferiority in more severe cases, we found it appropriate to set the score value ≥3 as a cut-off in the analysis of the PPC incidence [55].

The interventional part of our trial relied on two sets of facts. The first is that CS operations with ECC inevitably induce a complex, multi-etiological, phasic OS and SIR, which are the most intense within the first 48 h after CS, and which, in addition to the other factors, affect postoperative pulmonary function [1,2,3,4,5,12,13,14,15]. The second relates to the proven antioxidant and pleiotropic biochemical functions of the VitC, for which it has been commonly used to reduce organ damage induced by excessive OS and SIR [24,25,26,27,28,29,30,31,32,33].

About 63 million years ago, the primates and a few other species were “deceived” by the abundance of plants containing VitC, and conserved the mutation and inactivation of the gene for the synthesis of L-gulono-γ-lactone oxidase (GULO) on chromosome 8p21, thus losing the ability to synthesize VitC from glucose. Accordingly, humans entirely rely on the dietary intake and rational metabolic use of VitC to maintain homeostasis [70]. A recent study of VitC status and the prevalence of deficiency suggests that dietary intake is globally insufficient [71], a fact that seriously affects the clinical outcomes of surgical patients [29,34,35].

The first appreciation of the importance of VitC dietary intake came from British sailors. Upon the observation of naval surgeon James Lind (1747) that oranges and lemons can prevent scurvy (lat. Scorbutus), Gilbert Blane (1795) managed to persuade the Admiralty to use citrus juice as a daily ration on board British naval vessels [72]. Two centuries and two Nobel Prizes later (Albert Szent-Györgyi and Walter Norman Haworth), this low-cost, essential substance was the subject of many controversial polemics, sometimes surpassing purely medical and scientific frameworks [73]. Seneca, a Roman stoic philosopher, once said, “Truth never gets old”. Thus, as a result of accumulated knowledge of its important pleiotropic functions, VitC therapy has continued to be tested in numerous contemporary studies, mainly in critically ill patients.

Regardless of preoperative status, CS rapidly consumes VitC and serious depletion may last for two weeks, deteriorating the defense against OS and SIR during cardiac operations [25,29,34,35]. To our knowledge, there is no trial comparing the effectiveness of different therapeutic regimens (different doses and different duration of therapy) of parenterally administered VitC in CS patients. We aimed to test PPC in CS patients intervening with high daily parenteral doses of VitC (200 mg/kg/24 h) for the first 48 postoperative hours. Similar (i.e., 250 mg/kg), single parenteral doses were tested in two studies on CS patients, one aimed to compare the postoperative dynamics of creatine kinase-MB and malondialdehyde [56], and another to compare the dynamics of the cardiac index with a control group [45]. Both studies have reported the beneficial effect of VitC [45,56]. A trial with the same dose of parenteral VitC as ours, given for 96 h, in patients with severe sepsis, has proven its benefits in the dynamics of the inflammatory markers and SOFA score [44]. None of them have reported any adverse effects of high-dose parenteral VitC therapy [44,45,56]. We also did not have any adverse effects or complications with our VitC therapeutic regimen. To assess potential functional renal impairment induced by high doses of VitC, we included renal functional analysis in secondary outcome measures. Renal function was preoperatively significantly better in Group B, and this remained the same up to 96 h after CS (Table 1 and Table 3, Supplementary Materials S1, Figure S3).

The incidence and severity of PPC were significantly lower in Group B (see Table 2). These results are in concordance with the only relatively comparable published trial [55]. Comparing the PPC by grade, in Group A most of them were clustered in grades 2–4, while in Group B they clustered in grades 1 and 2 (see Table 2, Supplementary Materials S1, Figure S1).

The most common types of PPC in our trial were pleural effusion and pneumonia, all being significantly more frequent in Group A. Similar incidences are reviewed by Tanner et al. [62]. Grade 4 PPC (respiratory failure equivalent) was more frequent in Group A (see Table 2). Wang et al. reported only one (2.7%) patient, from the control group, with PPC grade 4. Such discrepancy may be explained by comparing the baseline perioperative characteristics. Patients in our trial were older and had advanced systemic disease, pronounced CVD risks, and serious comorbidities. Moreover, we had 26% of patients with a history of previous COVID-19 disease, while the trial of Wang et al. was conducted before the first cases of COVID-19 were reported in Wuhan, China, in December 2019 [55,60].

Parameters indicating pulmonary function (i.e., oxygenation and ventilation) and selected inflammatory markers, were sampled at 48 h after CS. The mean values of PaO_2_/FiO_2_ and A-aDO2 depict the presence of postoperative lung injury in both groups. A significantly better Horowitz index in Group B supports a potential lung protective effect of the VitC (see Table 3). In a trial by Wang et al. there was no difference in these parameters between the groups [55].

The median serum procalcitonin levels were not elevated as they are in septic patients but were significantly lower in Group A. At 48 h, there was no significant difference between the other inflammatory markers, except CRP and sedimentation rate, which were significantly lower in Group B (see Table 2). The dynamics of those parameters were followed up for 96 h. Interestingly, from 48 h to 96 h, statistically lower values were also recorded for leucocytes, neutrophils, and lymphocytes in Group B, and fibrinogen in Group A (Supplementary Materials S1, Figure S2). These findings support the potential anti-inflammatory effect of VitC which spans more than 48 h after CS.

Except for acute renal failure and wound infection, the other non-pulmonary complications did not show significant differences between the groups (see Table 3). Iizuka et al. found a positive correlation between low plasma levels of VitC and postoperative delirium in elderly CS patients [74], but this was not the case in our trial.

SOFA score, estimated 48 h after CS did not show a significant difference between the groups in our trial. Median values of four indicate a mild degree of organ dysfunction in all patients at this time frame. In other studies of high parenteral doses of VitC in critically ill patients, reviewed by Nabzdyk et al., a significant reduction in SOFA score grade was recorded in the intervention groups [75]. This difference may be interpreted in light of different underlying causes of organ dysfunction (i.e., severe sepsis and severe burns vs. CS).

Patients in Group B had significantly lower ICU re-admission rates and shorter ICU stays. Also, they had a significantly lower hospital mortality rate (see Table 3). These results are better than those reported by Wang et al. [55], probably depicting the more physiological regimen (i.e., higher doses and longer duration) of parenteral VitC supplementation in our trial.

This trial has some limitations. First, as a single-centered and single-blinded trial, it was difficult to avoid selection, observer, performance, and detection biases, affecting the validity and reliability of the trial. To minimize these potential biases, the trial was kept open only to three persons (the principal investigator and two informed assistants). The key trial intervention (infusion of VitC solution) was masked, as explained earlier, to all but the principal investigator and two informed assistants. Similarly, the assessment of the key outcome parameter (PPC score) and other scoring systems (ASA and SOFA) was protected from the influence of informed researchers by engaging two independent, blinded assistants. Second, although the calculated sample size of 70 participants in each group provides this trial with an acceptable level of statistical accuracy and validity, this number is still too small to draw any general conclusions, so the results of this trial should be interpreted with caution. Third, highly inconsistent criteria for the definition of PPC make the results of this, like other similar studies, insufficiently suitable for comparisons and meta-analyses. That was why we decided to use the modified [20,21,22] PPC severity score, originally defined by Kroenke et al. [23], as an outcome measure for our primary end-point. Our decision is supported by the fact that this score was also used in the only trial published so far on the effect of VitC on PPC in CS [55]. Fourth, setting the score value ≥3 as a cut-off in the analysis of the PPC incidence, we aimed to “catch” the more severe cases, and thus we probably “lost” some of the less severe PPC from the analysis of PCC incidence. Fifth, we could not measure plasma levels of the VitC, due to the complexity of the procedure and the excessive cost of the reagents. Because of the complex pharmacokinetics of VitC [30], it would be the most reliable way to interpret the observed differences between the control (A) and the intervention group (B), in the context of the possible influence of the applied high parenteral VitC doses. Finally, further multicenter, prospective, randomized, controlled, double-blinded, interventional trials, would (hopefully) confirm our results. Meanwhile, we are planning to conduct a new prospective, randomized, controlled study of multiple parallel groups to evaluate the effectiveness of four different perioperative therapeutic regimens of intravenous VitC on PPC in CS patients. Before that, it is necessary that a widely accepted, standardized, and comparable definition of PPC finally appears.

## 5. Conclusions

Our trial has shown that high parenteral daily VitC doses (200 mg/kg/24 h) could be given to selected CS patients during the first 48 h after CS without adverse effects or complications. By reducing the OS and SIR, along with many other pleiotropic functions, applied doses of VitC significantly reduce the incidence and severity of PPC in CS patients. In addition, patients receiving high parenteral daily VitC doses had significantly less damaged lungs and better postoperative renal function, shorter ICU stay, fewer ICU re-admissions, and lower hospital mortality. Further multicenter RCTs are needed to confirm these results.

## Figures and Tables

**Table 1:** Baseline perioperative characteristics.

Perioperative Parameters	Group A (*n* = 75)	Group B (*n* = 75)	*p*-Value (Test) *
***1. Demographic and Anthropometric***			
Age (years)	66.9 ± 8.7	66.3 ± 8.6	0.672 (t)
Male gender	59 (78.7%)	55 (73.3%)	0.444 (chi)
BMI (kg/m^2^)	28.4 ± 3.9	26.7 ± 3.3	0.005 (t)
***2. CVD Risk***			
HTA	75 (100%)	73 (97.3%)	0.497 (fet)
DM	36 (48%)	36 (48%)	1.000 (fet)
HLP	72 (96%)	68 (90.7%)	0.190 (chi)
Smoking	52 (69.3%)	52 (69.3%)	1.000 (chi)
***3. CV Status and Comorbidities***			
Recent MI	41 (54.7%)	34 (45.3%)	0.253 (chi)
AP	61 (81.3%)	58 (77.3%)	0.545 (chi)
TAs (mmHg)	145.8 ± 26.3	139.8 ± 16.9	0.100 (t)
TAd (mmHg)	81.9 ± 13.1	77.3 ± 11.6	0.022 (t)
EF-LV (%)	46.5 ± 9.1	48.4 ± 8.0	0.187 (t)
HR (beats/min)	70.4 ± 9.3	69.5 ± 9.7	0.565 (t)
Sinus	68 (90.7%)	66 (88%)	0.597 (chi)
AF	8 (10.7%)	9 (12%)	0.979 (chi)
CVD	11 (14.7%)	5 (6.7%)	0.113 (chi)
CRF	23 (30.7%)	12 (16%)	0.034 (chi)
COVID-19	22 (29.3%)	17 (22.7%)	0.352 (chi)
***4. Pulmonary status (PPC Score)***			
0	20 (26.7%)	25 (33.3%)	0.373 (chi)
1	55 (73.3%)	50 (66.7%)	
***5. ASA Score***			
3	65 (86.7%)	66 (88.0%)	0.806 (chi)
4	10 (13.3%)	9 (12.0%)	
***6. Surgery***			
CABG	53 (70.7%)	49 (65.3%)	0.484 (chi)
Aortic valve	9 (12%)	13 (17.3%)	0.356 (chi)
Mitral valve	2 (2.7%)	4 (5.3%)	0.681 (fet)
Combined	11 (14.7%)	9 (12%)	0.631 (chi)
Duration of surgery (min)	245.7 ± 40.2	219.9 ± 45.0	<0.001 (t)
ECC time (min)	86.8 ± 27.3	80.7 ± 19.1	0.114 (t)
ACC time (min)	55.6 ± 20.7	56.3 ± 15.4	0.799 (t)

Legend: BMI—Body mass index; HTA—Arterial hypertension; DM—Diabetes Mellitus; HLP—Hy-perproteinemia; CV—Cardio-vascular; MI—Myocardial infarction; AP—Angina pectoris; TAs—Systolic arterial pressure; TAd—Diastolic arterial pressure; EF-LV—Left ventricular ejection fraction; CVD—Cerebro-vascular diseases; CRF—Chronic renal failure; COVID-19—Coronavirus disease (SARS-CoV-2 virus); PPC—Postoperative pulmonary complications score; ASA—American Society of Anesthesiologists; CABG—Coronary artery bypass grafting; ECC—Extracorporeal circulation; ACC—Aortic cross-clamp. The results are presented as count (%) or mean ± sd. * Statistical tests: (t)—Student’s *t*-test; (chi)—Chi-Square (Χ^2^) Test; (fet)—Fisher’s exact test.

**Table 2:** Primary end-point outcome measures: PPCs incidence, severity, and types.

Primary Outcome Measures	Group A (*n* = 75)	Group B (*n* = 75)	*p*-Value (Test) *
***1. PPCs Incidence***			
PPC ≥ 3 (*n*, %)	45 (60.0%)	10 (13.3%)	<0.001 (chi)
***2. PPCs Severity***			
PPC severity score	3 (2)	1 (1)	<0.001 (mw)
Grade 0 (*n*, %)	5 (6.7%)	14 (18.7%)	<0.001 (mw)
Grade 1 (*n*, %)	3 (4.0%)	31 (41.3%)	
Grade 2 (*n*, %)	22 (29.3%)	20 (26.7%)	
Grade 3 (*n*, %)	23 (30.7%)	7 (9.3%)	
Grade 4 (*n*, %)	18 (24.0%)	3 (4.0%)	
Grade 5 (*n*, %)	4 (5.3%)	0	
***3. PPCs Types***			
Pneumonia (*n*, %)	32 (42.7%)	13 (17.3%)	<0.001 (chi)
Pneumothorax (*n*, %)	10 (13.3%)	3 (4%)	0.042 (chi)
Pleural effusion (*n*, %)	51 (68%)	38 (50.7%)	0.031 (chi)
Re-intubation (*n*, %)	16 (21.3%)	2 (2.7%)	<0.001 (chi)

Legend: PPC—Postoperative pulmonary complication. The results are presented as count (%), or median (IQR). * Statistical tests: (chi)—Chi-Square (Χ^2^) Test; (mw)—Mann–Whitney test.

**Table 3:** Secondary end-point outcome measures.

Secondary Outcome Measures	Group A (*n* = 75)	Group B (*n* = 75)	*p*-Value (Test) *
***1. Pulmonary oxygenation and ventilation***			
Horowitz index (PaO_2_/FiO_2_) 48 h	268.9 ± 112.6	312.6 ± 107.4	0.008 (t)
Alveolar–arterial gradient (A-aDO_2_) 48 h	17.3 ± 5.4	17.9 ± 4.7	0.432 (t)
Total MV time (h)	5.2 ± 1.6	5.4 ± 1.2	0.493 (t)
2. Inflammatory markers (48 h) **			
Procalcitonin	0.3 (0.6)	0.5 (0.8)	0.032 (mw)
C-reactive protein	167.4 (82.9)	95 (56.2)	<0.001 (mw)
Leucocytes	13.3 ± 3.3	12.4 ± 3.4	0.102 (t)
Neutrophils	80.6 ± 5.5	80.5 ± 5.1	0.881 (t)
Lymphocytes	12 ± 4.6	12.6 ± 4	0.418 (t)
Sedimentation rate	22 (18)	20 (6)	0.023 (mw)
Fibrinogen	5.1 ± 1.4	5.2 ± 1	0.775 (t)
Albumin	31.8 ± 3.5	31.4 ± 3.2	0.479 (t)
D-dimer	0.5 (0.5)	0.6 (0.4)	0.877 (mw)
Ferritin	202 (277)	200 (104)	0.287 (mw)
***3. Postoperative complications (non-pulmonary)***			
PONV	20 (26.7%)	30 (40.0%)	0.083 (chi)
Delirium	22 (29.3%)	16 (21.3%)	0.260 (chi)
Transfusion	32 (42.7%)	36 (48%)	0.512 (chi)
Acute renal failure	8 (10.7%)	1 (1.3%)	0.034 (fet)
Wound infection	15 (20%)	5 (6.7%)	0.016 (chi)
CPR	4 (5.3%)	0 (0%)	0.120 (fet)
4. Renal function (48 h) **			
GFR < 60 mL/min	24 (32%)	10 (13.3%)	0.006 (chi)
Creatinine	99.6 ± 44.6	87.8 ± 27.5	0.054 (t)
Urea	7.1 ± 3	6.3 ± 1.9	0.041(t)
***5. Postoperative organ dysfunction***			
SOFA score	4 (2)	4 (1)	0.132 (mw)
ASA/SOFA ratio	1.0 (0.4)	0.8 (0.25)	0.190 (mw)
***6. ICU outcome measures***			
ICU re-admission	15 (20%)	4 (5.3%)	0.007 (chi)
ICU stay	48 (24)	32 (24)	<0.001 (mw)
***7. Hospital outcome measures***			
Hospital stay	8 (2)	8 (2)	0.092 (mw)
Hospital mortality	8 (10.7%)	1 (1.3%)	0.034 (fet)

Legend: MV—Mechanical ventilation; PONV—Postoperative nausea and vomiting; CPR—Cardio-pulmonary resuscitation; GFR—Glomerular filtration rate; SOFA—Sequential Organ Failure Assessment, ASA—American Society of Anesthesiologists; ICU—Intensive care unit. The results are presented as count (%), mean ± sd, or median (IQR). * Statistical tests: (t)—Student’s *t*-test; (mw)—Mann–Whitney test; (chi)—Chi-Square (Χ^2^) Test; (fet)—Fisher’s exact test. ** Dynamics of inflammatory markers, measured at postoperative 0 h, 24 h, 48 h, 72 h, and 96 h time frames, available in Supplementary Materials S1, Figure S2, and for the GFR < 60 mL/min, in Supplementary Materials S1, Figure S3.

## Data Availability

The deidentified participant data and data dictionaries from this trial will be available upon request to researchers who provide a methodologically sound proposal for analyses that are in line with the original study objectives. The data will be available from 6 months after publication until 3 years following the publication of the study. To gain access, data requestors will need to sign a data access agreement and agree to report their findings in a peer-reviewed journal. Data will be shared through a secure online platform. The study protocol, statistical analysis plan, informed consent form, and clinical study report will be available on the trial website.

## Supplementary Materials

The following supporting information can be downloaded at: https://www.mdpi.com/article/10.3390/nu16060761/s1, Supplementary Materials S1: Table S1: PPC severity score; Figure S1: PPC by grade; Figure S2: Dynamics of inflammatory markers; Figure S3: Dynamics of GFR < 60 mL/min; Figure S4: Regimen of VitC administration. Supplementary Materials S2: Consort 2010 Flow Diagram. Supplementary Materials S3: Consort 2010 Flow Check-List.
