# Supplementary material for: The Influence of High-Dose Parenteral Vitamin C on the Incidence and Severity of Postoperative Pulmonary Complications in Cardiac Surgery with Extracorporeal Circulation: A Randomized Controlled Trial
Source: Nutrients. 2024 Mar 7;16(6):761. doi: 10.3390/nu16060761 (PMC10975872; doi:10.3390/nu16060761)
Supplement: Supplementary file 1 [file nutrients-16-00761-s001.zip › Su pplementary Materials S1.pdf]

Supplement 1

Table S1. PPCs severity score (modified from references in text [20–23,58].

PPC Score	Operational Definitions
**Grade 0**	No PPC
**Grade 1**<br>(1 of the items)	• a dry cough<br>• micro atelectasis: abnormal auscultatory findings and temperature ≥ 37.5°C without another documented cause with normal X-ray of the lungs<br>• dyspnea without another documented cause
**Grade 2**<br>(2 of the items)	• productive cough without another documented cause<br>• bronchospasm - new wheezing or old wheezing that requires a change in therapy<br>• hypoxemia in room air ($SpO_2 \leq 90\%$)<br>• radiologically confirmed atelectasis (two independent experts) plus: temperature >37.5°C or abnormal auscultatory findings<br>• hypercarbia requiring treatment ($PaCO_2$>50 mm Hg)
**Grade 3**<br>(1 of the items)	• pleural effusion requiring thoracentesis<br>• pneumonia: radiological confirmation (two independent experts) plus clinical symptoms (two of the following: leukocytosis or leukopenia, abnormal temperature, purulent discharge) plus isolation of the causative agent (G+ or G- culture) or necessary change of antibiotic therapy<br>• pneumothorax<br>• prolonged non-invasive ventilation due to all three reasons: a) $SpO_2 \leq 92\%$ on oxygen supplementation, b) oxygen supplementation >5L, c) RR≥30 bpm<br>• postoperative intubation or reintubation, i.e. dependence on mechanical ventilation (invasive or non-invasive) lasting less than 48 hours
**Grade 4**	Ventilatory insufficiency: postoperative dependence on mechanical ventilation for more than 48 hours or reintubation with subsequent dependence on mechanical ventilation for more than 48 hours
**Grade 5**	Death before hospital discharge

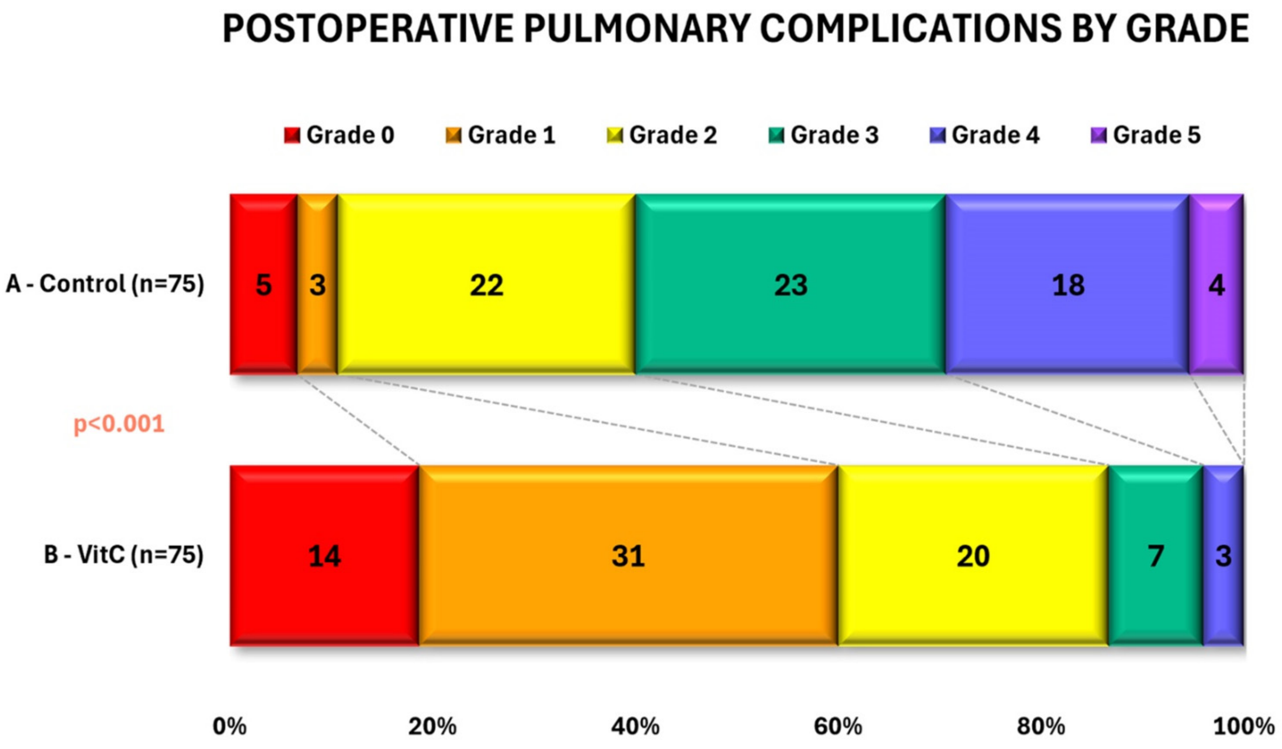

Figure S1. PPCs by grade.

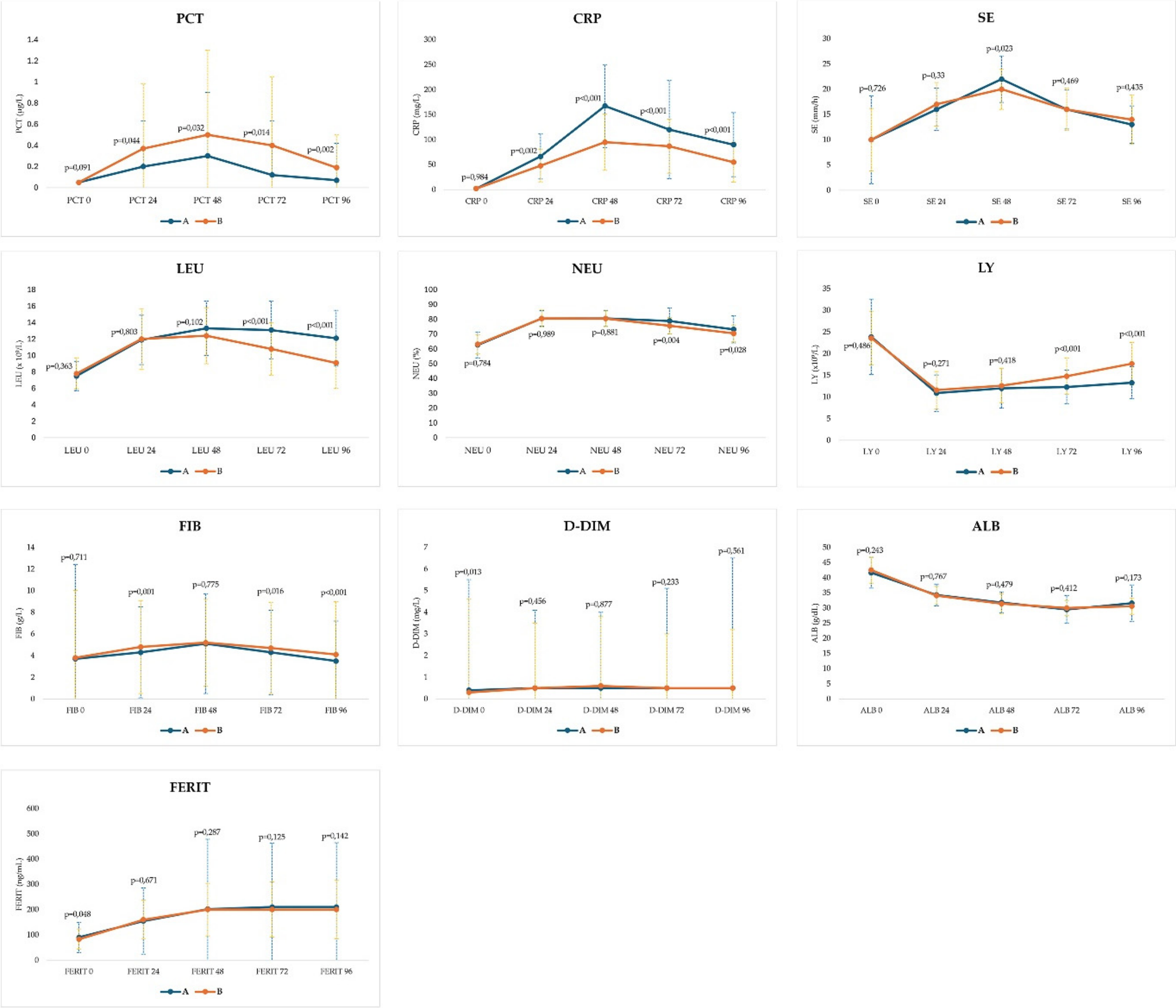

Figure S2. Dynamics of inflammatory markers.

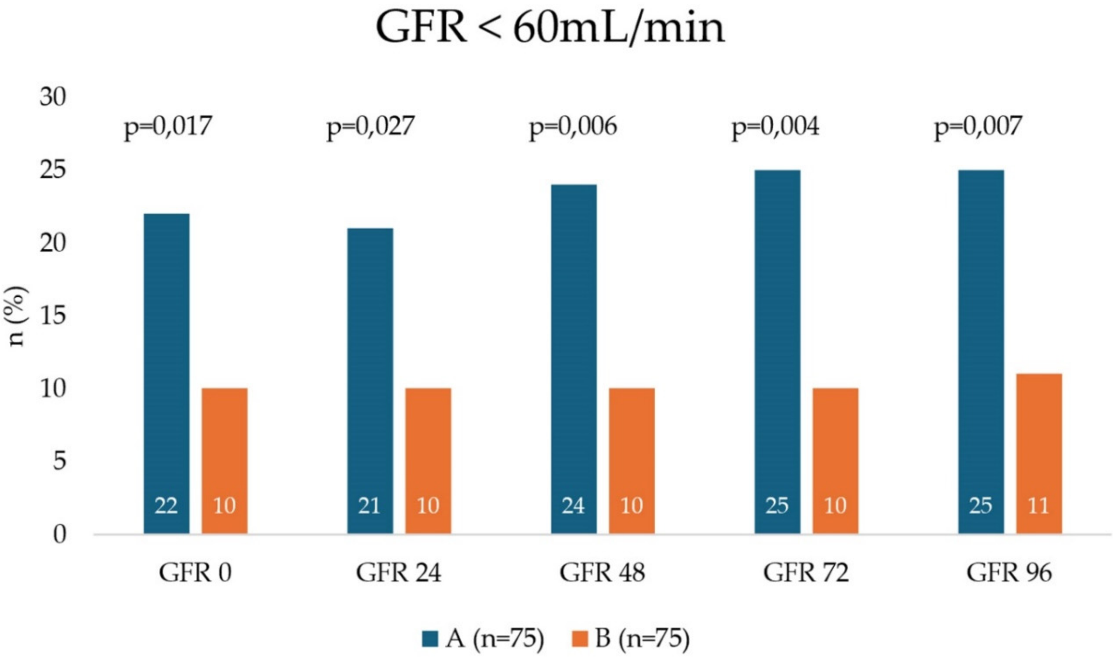

Figure S3. Dynamics of GFR < 60mL/min.

A

O.D. (0-24h) — **Control : normal saline**

I dose (intraoperatively): equal volume of normal saline divided in 3 equal parts and given:

1. 10 min after induction of anesthesia
2. 10 min before ACC removal
3. At the beginning of sternal closure

II dose (6 h after I intraoperative dose): equal volume of normal saline
III dose (6 h after II dose): equal volume of normal saline
IV dose (6 h after III dose): equal volume of normal saline

P.O.D. 1 (24-48h) — **Control : normal saline**

V dose (6 h after IV dose): equal volume of normal saline
VI dose (6 h after V dose): equal volume of normal saline
VII dose (6 h after VI dose): equal volume of normal saline
VIII dose (6 h after VII dose): equal volume of normal saline

B

O.D. (0-24h) — **Daily dose: 200mg/kg, Q6h (i.e. single dose 50mg/kg)**

I dose (intraoperatively): 50 mg/kg divided into 3 equal parts, each diluted in 10 ml of normal saline and given:

1. 10 min after induction of anesthesia
2. 10 min before ACC removal
3. At the beginning of sternal closure

II dose (6 h after I intraoperative dose): 50 mg/kg diluted in 50 ml of normal saline
III dose (6 h after II dose): 50 mg/kg diluted in 50 ml of normal saline
IV dose (6 h after III dose): 50 mg/kg diluted in 50 ml of normal saline

P.O.D. 1 (24-48h) — **Daily dose: 200mg/kg, Q6h (i.e. single dose 50mg/kg)**

V dose (6 h after IV dose): 50mg/kg diluted in 50 ml of normal saline
VI dose (6 h after V dose): 50 mg/kg diluted in 50 ml of normal saline
VII dose (6 h after VI dose):50 mg/kg diluted in 50 ml of normal saline
VIII dose (6 h after VII dose):50 mg/kg diluted in 50 ml of normal saline

Figure S4. Regimen of VitC administration.
