# Supplementary figures and images for: The Influence of High-Dose Parenteral Vitamin C on the Incidence and Severity of Postoperative Pulmonary Complications in Cardiac Surgery with Extracorporeal Circulation: A Randomized Controlled Trial
Source: Nutrients. 2024 Mar 7;16(6):761. doi: 10.3390/nu16060761 (PMC10975872; doi:10.3390/nu16060761)

## CONSORT 2010 Flow Diagram

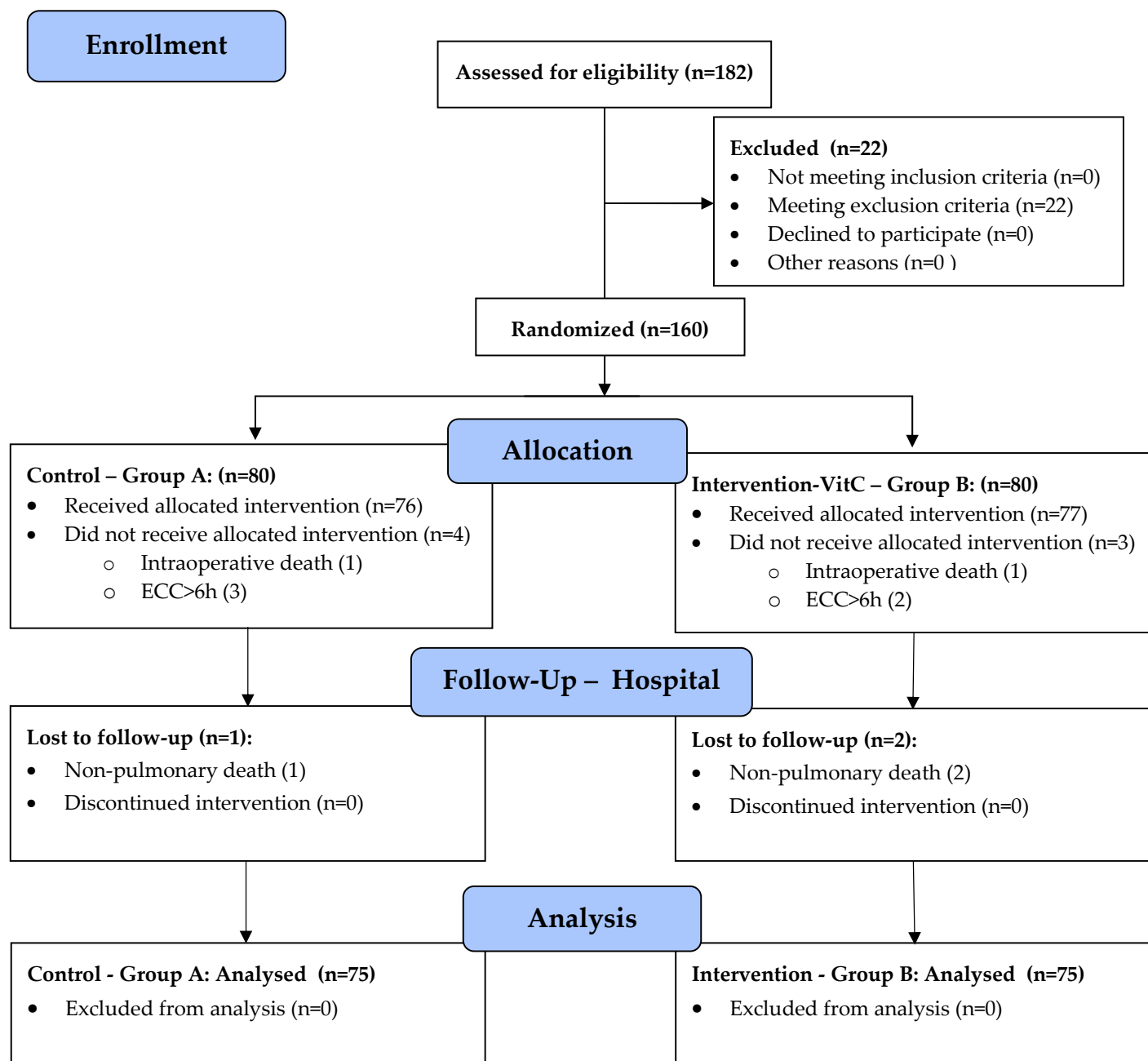

Supplement: Supplementary file 1 [file nutrients-16-00761-s001.zip › Su pplementary Materials S2. Consort 2010 Flow Diagram.pdf]
